# Effects of discontinuing or continuing ongoing statin therapy in severe sepsis and septic shock: a retrospective cohort study

**DOI:** 10.1186/cc10317

**Published:** 2011-07-18

**Authors:** Armand Mekontso Dessap, Islem Ouanes, Nerlep Rana, Beatrice Borghi, Christophe Bazin, Sandrine Katsahian, Anne Hulin, Christian Brun-Buisson

**Affiliations:** 1 AP-HP, Groupe Henri-Mondor Albert-Chenevier, Service de Reanimation Medicale, 51 avenue du Mal de Lattre de Tassigny Creteil, F-94010 France; 2 INSERM, Unite U955, 51 avenue du Mal de Lattre de Tassigny, Creteil, F-94010, France; 3 Faculté de Medecine, Universite Paris 12, 8 avenue du Général Sarrail, Creteil, F-94010, France; 4 Intensive Care Unit, Fattouma Bourguiba University Hospital, avenue Farhat HACHED, 5000 Monastir, Tunisia; 5 AP-HP, Groupe Henri-Mondor Albert-Chenevier, Laboratoire de Pharmacologie et Toxicologie, 51 avenue du Mal de Lattre de Tassigny, Creteil, F-94010 France; 6 AP-HP, Groupe Henri-Mondor Albert-Chenevier, Unité de Reherche Clinique, 51 avenue du Mal de Lattre de Tassigny, Creteil, F-94010 France

**Keywords:** statin, discontinuation, blood concentration, sepsis

## Abstract

**Introduction:**

Recent publications suggest potential benefits from statins as a preventive or adjuvant therapy in sepsis. Whether ongoing statin therapy should be continued or discontinued in patients admitted in the intensive care unit (ICU) for sepsis is open to question.

**Methods:**

We retrospectively compared patients with severe sepsis and septic shock in whom statin therapy had been discontinued or continued. The primary endpoint was the number of organ failure-free days at day 14. Secondary end-points included hospital mortality and safety. The association of statin continuation with outcome was evaluated for crude analysis and after propensity score matching and adjustment. We also measured plasma atorvastatin concentrations in a separate set of ICU septic patients continuing the drug.

**Results:**

Patients in whom statin therapy had been continued in the ICU (n = 44) had significantly more organ failure-free days (11 [6-14] vs. 6 [0-12], mean difference of 2.34, 95%CI from 0.47 to 5.21, *P *= 0.03) as compared to others (n = 32). However, there were important imbalances between groups, with more hospital-acquired infections, more need for surgery before ICU admission, and a trend towards more septic shock at ICU admission in the discontinuation group. The significant association of statin continuation with organ failure free days found in the crude analysis did not persist after propensity-matching or multivariable adjustment: beta coefficients [95% CI] of 2.37 [-0.96 to 5.70] (*P *= 0.20) and 2.24 [-0.43 to 4.91] (*P *= 0.11) respectively. We found particularly high pre-dose and post-dose atorvastatin concentrations in ICU septic patients continuing the drug.

**Conclusions:**

Continuing statin therapy in ICU septic patients was not associated with reduction in the severity of organ failure after matching and adjustment. In addition, the very high plasma concentrations achieved during continuation of statin treatment advocates some caution.

## Introduction

Statins are effective lipid-lowering agents that have been shown to improve survival in the primary and secondary prevention of atherosclerosis in several large randomized clinical trials [1]. Many experimental models have also shown pleiotropic activity of statins (including anti-inflammatory, anti-oxidative, and immunomodulatory effects) that may account for a potential beneficial impact during sepsis [2,3]. A recent systematic review and meta-analysis of 20 clinical studies suggests that statins may have a positive impact on the outcome of patients with infection or sepsis [4]. Since January 2006 [2], we encouraged continuation of ongoing statin therapy whenever possible in patients chronically treated with statins who were admitted to our ICU with severe sepsis, although current prescribing guidelines still suggest caution in the continued use of statins in patients hospitalized for acute illness because of concern of serious side effects [5].

The aim of this preliminary report was: to evaluate the effectiveness and safety of statin therapy continuation on the incidence of organ failure in septic patients (compared with patients in whom statins were routinely stopped); and to assess atorvastatin plasma concentrations during its continuation in a subset of ICU septic patients.

## Materials and methods

The study was approved by the institutional ethics committee of the "Société de Réanimation de Langue Française". Informed consent was waived and written and oral information about the study was given to the families.

### Patients

We conducted a retrospective cohort study among patients admitted between January 2005 and August 2007 for severe sepsis and septic shock in our ICU and with ongoing statin therapy (initiated at least one month before ICU admission and continued with no interruption until ICU admission). Severe sepsis or septic shock was defined according to the ACCP/SCCM (American College of Chest Physicians/Society of Critical Care Medicine) Consensus Conference [6]. Non-inclusion criteria included a moribund state, an anticipated ICU stay of less than 24 hours, a contraindication to enteral statin therapy administration (intolerance to enteral feeding with vomiting), liver dysfunction with aminotransferase enzymes (either aspartate or alanine) more than three times the upper limit of normal (ULN), rhabdomyolysis with creatine phosphokinase (CPK) levels above five ULN, myopathy, status epilepticus, concomitant administration of azole derivatives, delavirdine, or telithromycin.

### Discontinuation and continuation groups

In the first period (January to December 2005), routine discontinuation of ongoing statin therapy at admission of septic patients to our ICU was recommended. The second period (January to December 2006) was an overlap period during which the decision to continue or discontinue ongoing statin therapy was left to the clinician in charge of the patient. During the third period (January to August 2007), routine continuation of ongoing statin therapy was encouraged. Consecutive patients with severe sepsis and septic shock were retrospectively identified from January 2005 to August 2007 using the unit computerized database and data collected by manual chart review. Two groups of septic patients were distinguished: the discontinuation group (patients in whom statins were stopped at ICU admission); and continuation group (patients in whom statins were continued whenever possible). Patients in both groups were managed in the ICU according to current recommendations [7].

### Statin continuation protocol

"Continuation" patients received their usual statin therapy orally (or via an oro-gastric tube, after crushing the tablets) in the same dosage as used during ambulatory care, while conforming to several other precautions as follows: i) the association with certain medications was avoided (oral anticoagulants, fibrates and cyclosporine for all statins, erythromycin, clarithromycin, or verapamil for atorvastatin and simvastatin); ii) if the patient received enteral feeding via an oro-gastric tube, gastric residue evaluations were performed at least six hours after drug administration. Possible side effects of statins were monitored, by reviewing daily blood chemistry as well as aminotransferase and CPK levels when available. Statin therapy was interrupted in the following cases: food intolerance with vomiting, aminotransferase elevation above three ULN, increase in serum CPK levels to above five ULN. In case of interruption of statin therapy because of food intolerance, the treatment was reintroduced after 24 hours of resumption of enteral feeding without vomiting. The analysis was performed on an intention-to-treat basis (i.e., patients in the continuation group remained in that group even if statins were secondarily discontinued during ICU stay).

### Study of atorvastatin plasma concentrations

We prospectively assessed atorvastatin pharmacokinetics during its continuation (in accordance with the above mentioned statin continuation protocol) in nine ICU patients admitted for severe sepsis or septic shock during the year 2008 (these patients are not included in the above mentioned retrospective cohort analysis). A total of 11 daily administrations of 40 mg of atorvastatin were assessed for plasma concentrations (two patients were assessed twice). Blood samples were collected pre-dose and at 90 minutes post-dose. Demographic data and information on concurrent medications that might interact with the cytochrome P450 3A4 enzyme system were collected. After centrifugation, plasma was stored frozen at -80°C until analysis. Samples were analysed by High Performance Liquid Chromatography (ThermoFisher Scientific, Waltham, USA) using ultraviolet detection at 245 nm (Waters, Saint-Quentin, France) [8]. The mobile phase was NaH_2_PO_4 _10 mM pH5.5/ACN (67/33, v/v), and thiopental was used as internal standard. Standard calibration curves for atorvastatin were linear over concentrations ranging from 20 to 200 ng/mL (average r^2 ^of 0.99). The limit of quantification was 20 ng/mL. Intraday precision was good with a coefficient of variation of 15.3%, 6.0%, and 3.2% for three levels of control 25, 75, and 150 ng/mL. Interday precision was also acceptable with coefficient of variation of 18%, 7.1%, and 5.7%, respectively. Accuracy was correct with error percentages of 5.1%, 2.0%, and 2.5%, respectively, for the three controls.

### Endpoints

The primary endpoint was the number of organ failure-free days up to day 14. Secondary end-points included the number of hemodynamic failure-free days and organ dysfunction-free days up to day 14, ICU and hospital survival, and evaluation of treatment safety, assessed as the proportion of patients with serum CPK above three ULN and of patients with a transaminases level above two ULN. Organ dysfunction and organ failure were defined by a Sequential Organ Failure Assessment (SOFA) score for the appropriate function above one and above two, respectively [9]. Hemodynamic failure was defined by a cardiovascular SOFA score above two (dopamine > 5 μg/kg/min, norepinephrine regardless of the dose and adrenaline regardless of the dose) [10]. Organ failure-free days were defined as the number of days between ICU admission (day 1) and day 14 with the patient alive without any organ failure. Organ failure-free days were considered equal to zero in case of ICU death before day 14. Patients with unavailable SOFA score (because of ICU discharge before day 14) were considered free from organ failure after ICU discharge. We also assessed the pre-dose and post-dose plasma atorvastatin concentrations during its continuation.

### Statistical analysis

Statistical analysis was performed using SPSS Base 17.0 package (SPSS Inc, Chicago, IL, USA) and the nonrandom package 1.1 using R 2.10.1 (The R Foundation for Statistical Computing, Vienna, Austria) [11]. Dichotomous variables are reported as percentage and compared using the Chi-square test or exact Fisher test (when the expected count was < 5). Quantitative variables are reported as median (1^st ^quartile to 3^rd ^quartile) and compared using the nonparametric Mann-Whitney test. In addition, we used standardized differences to estimate the balance in measured variables between continuation and discontinuation groups, independently of the sample size and variable unit [12].

We used propensity score analyzes to better scrutinize the association of statin continuation with the primary outcome (organ failure free days). The rationale and methods underlying the use of propensity scores for proposed causal exposure variables have been previously described [13,14]. The selection of covariates included in the multivariable logistic regression model used to estimate the propensity score for statin continuation was guided by clinical significance and imbalances between continuation and discontinuation groups, as estimated by an absolute standardized difference above 20% and/or a relative effect above five (relative effect is a measure describing the extent to which a covariate is confounding the effect of statin continuation on outcome). The final propensity score model included the following covariates: simplified acute physiology score (SAPS) II score at ICU admission, SOFA score at ICU admission, prior statin therapy duration, surgery treatment before ICU admission, septic shock at ICU admission, infection site, causative organism, type of infection (community acquired vs. hospital acquired), low-dose corticosteroid treatment, and fiscal year of ICU admission (in order to consider potential ongoing temporal changes in sepsis-related outcomes in our unit). We matched patients with statin continuation to those with statin discontinuation, using a greedy-matching algorithm with a calliper width of 0.2 standard deviations of the log odds of the estimated propensity score and sampling without replacement [15]. We used a graphical representation with standardized differences to check the balance of covariates in the matched sample [14]. In addition to propensity score matching, we performed a direct adjustment for confounding using a traditional linear regression model, with the same items selected for the propensity score as covariates and organ failure free days as the dependent variable [16]. A *P *value less than 0.05 was considered significant in bilateral analysis.

## Results

### Patients

**Figure 1:**
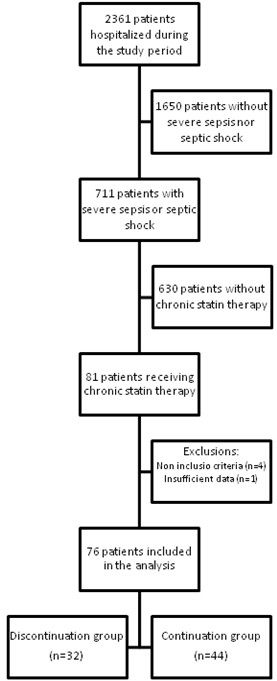
**Flow-chart of the study population**.

During the study-period, 81 patients receiving chronic statin therapy were hospitalized for severe sepsis or septic shock in our ICU. Five patients were excluded from the study because of non-inclusion criteria at ICU admission (vomiting in two patients and rhabdomyolysis in two others) or insufficient data (one patient; Figure 1). Statins were discontinued upon admission to the ICU in 32 patients (17 in 2005 and 15 in 2006, none in 2007) and continued in 44 patients (7 in 2005, 23 in 2006, and 14 in 2007). Table 1 and Table 2, respectively, display patients' and infection characteristics at ICU admission. Patients in the discontinuation group had significantly more hospital-acquired infections, more need for surgery before ICU admission, and a trend towards more septic shock at ICU admission as compared with the continuation group.

**Table 1:** Characteristics of 76 patients with severe sepsis and septic shock according to discontinuation or not of ongoing statin therapy at ICU admission

	Discontinuation group (n = 32)	Continuation group (n = 44)	*P *value	Absolute standardized difference* (%)
Age, years	71.1 (61.7-78.7)	72.9 (62.3-79.6)	0.66	7.7
Male gender, n (%)	26 (81%)	33 (75%)	0.52	15.0
SAPS II at ICU admission	44 (34-62)	40 (32-51)	0.15	37.5
SOFA at ICU admission	8 (5-10)	7 (4-9)	0.21	31.0
Mac Cabe classification,** n (%)			0.13	6.8
Nonfatal or no underlying disease	15 (47%)	18 (41%)		
Ultimately fatal underlying disease	13 (41%)	25 (57%)		
Rapidly fatal underlying disease	4 (13%)	1 (2%)		
Prior statin therapy duration, years (n = 67)	3.5 (1.0-6.3)	5.0 (1.4-7.6)	0.26	31.5
Type of statin, n (%)			0.28	15.4
Atorvastatin	13 (41%)	18 (41%)		
Pravastatin	9 (28%)	15 (34%)		
Simvastatin	10 (31%)	7 (16%)		
Rosuvastatin	0 (0%)	3 (7%)		
Fluvastatin	0 (0%)	1 (2%)		
Surgery before ICU admission, n (%)	11 (34%)	5 (11%)	0.02	56.1
Septic shock at ICU admission, n (%)	19 (59%)	16 (34%)	0.05	46.7

SAPS, simplified acute physiology score; SOFA, sequential organ failure assessment. *[12] **[32].

**Table 2:** Infection characteristics of 76 patients with severe sepsis and septic shock according to whether ongoing statin therapy was maintained or not at ICU admission

	Discontinuation group (n = 32)	Continuation group (n = 44)	*P *value	Absolute standardized difference* (%)
Infection site, n (%)			0.74	13.5
Lung	14 (44%)	17 (39%)		
Urinary tract	5 (16%)	7 (16%)		
Bloodstream (primary)	4 (13%)	7 (16%)		
Others	6 (19%)	5 (11%)		
Unknown	3 (9%)	8 (18%)		
Causative organism, n (%)			0.19	50.5
Gram-positive cocci				
*Staphylococcus *sp.	2 (6%)	3 (7%)		
*Streptococcus *and *Enterococcus *sp.	4 (13%)	7 (16%)		
Gram-negative bacilli				
Enterobacteriaceae sp	7 (22%)	13 (30%)		
Other Gram-negative bacilli	4 (13%)	6 (14%)		
*Other organisms*	0 (0%)	2 (5%)		
Polymicrobial	12 (38%)	5 (11%)		
Negative culture	3 (9%)	8 (18%)		
Type of infection, n (%)			<0.01	65.5
Community acquired	11 (34%)	29 (66%)		
Hospital acquired	21 (66%)	15 (34%)		
Adequation of initial antibiotic therapy, n (%)	25 (78%)	34 (77%)	0.93	2.0
Low-dose corticosteroids, n (%)	19 (59%)	18 (41%)	0.11	37.1
Activated protein C, n (%)	2 (6%)	0 (0%)	0.09	35.9

*[12]

### Outcomes

Patient outcomes in the entire cohort are reported in Table 3. The numbers of organ failure-free, hemodynamic failure-free, and organ dysfunction-free days were significantly higher in the continuation group as compared with the discontinuation group. The need for invasive mechanical ventilation and prevalence of acute respiratory distress syndrome were also significantly higher in the discontinuation group as compared with the continuation group. There was a trend towards increased hospital mortality and hospital length of stay in the discontinuation group as compared with the continuation group.

**Table 3:** Outcome of 76 patients with severe sepsis and septic shock according to discontinuation or not of ongoing statin therapy at ICU admission

	Discontinuation group (n = 32)	Continuation group (n = 44)	*P *value	Odds ratio (95% CI)
Invasive mechanical ventilation, n (%)	23 (72%)	21 (48%)	0.03	0.36 (0.14-0.94)
Duration of invasive mechanical ventilation, days	11 (4-18)	6 (3-20)	0.56	
Acute Respiratory Distress Syndrome, n (%)	13 (41%)	8 (18%)	0.03	0.33 (0.12-0.92)
Septic shock during ICU stay, n (%)	24 (75%)	23 (52%)	0.04	0.37 (0.14-0.99)
Organ failure free days*	6 (0-12)	11 (6-14)	0.03	
Organ dysfunction free days*	2 (0-10)	10 (3-12)	0.03	
Hemodynamic failure free days *	11 (7-13)	14 (10-14)	0.04	
ICU length of stay, days (ICU survivors, n = 57)	9 (4-16)	6 (4-10)	0.32	
Hospital length of stay, days (hospital survivors, n = 54)	36 (18-54)	24 (12-44)	0.09	
ICU deaths, n (%)	10 (31%)	9 (21%)	0.28	0.57 (0.20-1.61)
Hospital deaths, n (%)	13 (41%)	9 (21%)	0.06	0.38 (0.14-1.04)

CI, confidence interval.

*Organ failure free days, organ dysfunction free days, and vasoactive drug free days are calculated at day 14; Organ dysfunction was defined by a sequential organ failure assessment (SOFA) score above one for the appropriate function; Organ failure was defined by a SOFA score above two for the appropriate function.

**Figure 2:**
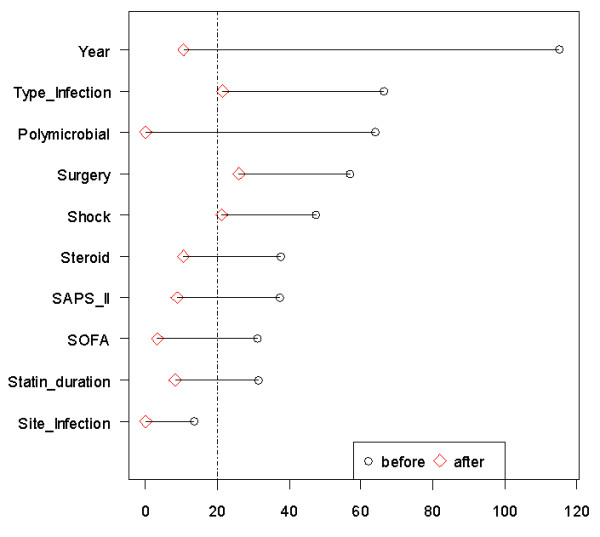
**Graphical representation of absolute standardized differences before and after propensity score matching comparing covariate values**. Imbalance for all variables was substantially reduced after matching. The 20% cut-off was used to select variables included in the propensity score. SAPS, simplified acute physiology score; SOFA, sequential organ failure assessment.

**Figure 3:**
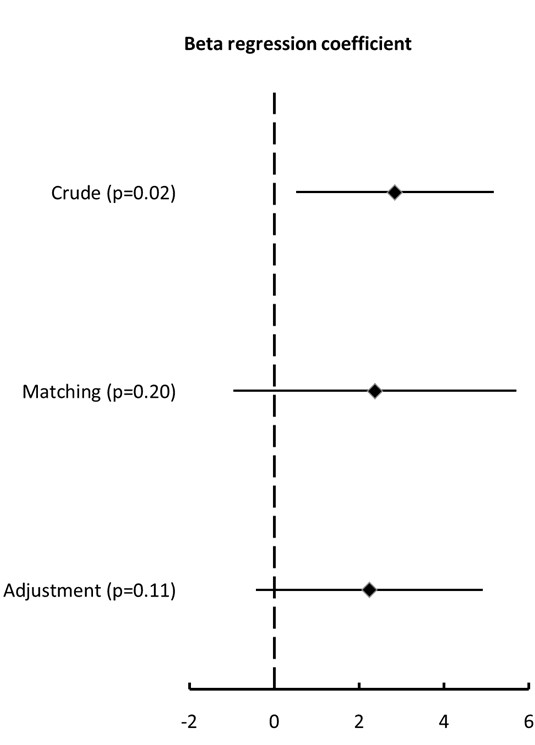
**Beta regression coefficients with 95% confidence intervals for the association between statin continuation and organ failure free days in models with crude analysis (2.84 (0.51 to 5.17), *P *= 0.02), propensity matching (2.37 (-0.96 to 5.70), *P *= 0.20) or multivariable adjustment (2.24 (-0.43 to 4.91), *P *= 0.11)**. The beta regression coefficient indicates the difference in mean number of days between continuation and discontinuation arms.

Of the 44 patients who continued statin therapy, 43% (19) were matched using the propensity score to a similar patient in whom statins were discontinued. The covariate balance between the continuation and discontinuation groups improved substantially through propensity-score matching (Figure 2). The association of statin continuation with organ failure-free days was not significant with the propensity-score matching (Table 4 and Figure 3) or with the linear regression adjustment (Table 5 and Figure 3).

**Table 4:** Outcome of patients with severe sepsis and septic shock according to discontinuation or not of ongoing statin therapy in the propensity-matched sample

	Discontinuation group (n = 19)	Continuation group (n = 19)	*P *value	Odds ratio (95% CI)
Invasive mechanical ventilation, n (%)	13 (68%)	10 (53%)	0.32	0.51 (0.14-1.92)
Duration of invasive mechanical ventilation, days	11 (4-18)	6 (4-31)	0.84	
Acute Respiratory Distress Syndrome, n (%)	8 (42%)	3 (16%)	0.07	0.26 (0.06-1.19)
Septic shock during ICU stay, n (%)	12 (63%)	13 (68%)	0.72	1.26 (0.33-4.84)
Organ failure free days*	6 (0-12)	9 (6-12)	0.23	
Organ dysfunction free days*	1 (0-9)	8 (3-11)	0.11	
Hemodynamic failure free days *	11 (7-14)	12 (7-14)	0.65	
ICU length of stay, days (ICU survivors, n = 27)	10 (4-17)	6 (5-10)	0.42	
Hospital length of stay, days (hospital survivors, n = 25)	29 (14-51)	20 (13-37)	0.45	
ICU deaths, n (%)	5 (26%)	6 (32%)	0.72	1.29 (0.32-5.28)
Hospital deaths, n (%)	7 (37%)	6 (32%)	0.73	0.79 (0.21-3.03)

CI, confidence interval.

* Organ failure free days, organ dysfunction free days, and vasoactive drug free days are calculated at day 14; Organ dysfunction was defined by a sequential organ failure assessment (SOFA) score above one for the appropriate function; Organ failure was defined by a SOFA score above two for the appropriate function.

**Table 5:** Multivariable linear regression model for organ failure free days

	Coefficient (ß)	Standard error	95% confidence interval	**Wald χ**^**2**^	*P *value
Intercept	2589	1832			
Statin continuation	2.24	1.36	(-0.43, 4.91)	2.71	0.10
SAPS II score at ICU admission	-0.10	0.03	(-0.16, -0.04)	9.26	<0.01
SOFA score at ICU admission	-0.37	0.27	(-0.90, 0.15)	1.93	0.17
Prior statin therapy duration	0.04	0.17	(-0.29, 0.36)	0.05	0.83
Surgery treatment before ICU admission	-1.30	1.64	(-4.52, 1.92)	0.62	0.43
Septic shock at ICU admission	1.90	1.75	(-1.53, 5.32)	1.18	0.28
Infection site	0.46	0.38	(-0.28, 1.19)	1.48	0.22
Polymicrobial infection	-3.46	1.55	(-6.49, -0.43)	5.01	0.03
Hospital-acquired infection	1.17	1.41	(-1.59, 3.94)	0.69	0.41
Low-dose corticosteroid treatment	-2.04	1.26	(-4.51, 0.44)	2.60	0.11
Year of ICU admission	-1.29	0.91	(-3.08, 0.51)	1.98	0.17

SAPS, simplified acute physiology score; SOFA, sequential organ failure assessment.

### Safety of statin continuation

Two patients in the continuation group required cessation of enteral diet and statin administration for 48 hours because of food intolerance with vomiting. Multiple blood concentrations of CPK and aminotransferases were available in 55 (72%) and 63 (83%) patients, respectively (representing 27% and 43% of patient-days in ICU for the continuation group, respectively). The proportion of patients with rhabdomyolysis (CPK levels increase above five ULN) or increase of liver enzymes (aminotransferases increase above three ULN) did not differ between the discontinuation and continuation groups: 3 (14%) vs. 1 (3%), *P *= 0.15; and 6 (25%) vs. 7 (18%), *P *= 0.54, respectively.

### Atorvastatin plasma concentrations during treatment continuation

**Figure 4:**
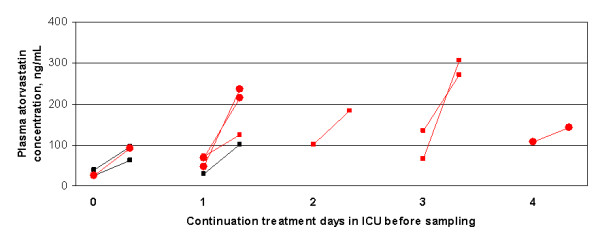
**Atorvastatin plasma concentrations before and 90 minutes after receiving a 40 mg dose in nine critically ill septic patients continuing this drug while under cytochrome P450 3A4 inhibitors (in red) or not (in black)**. Two patients were assessed twice (circles). The residual concentrations reported in healthy volunteers after a single 20 mg dose are close to 3 ng/mL [26].

We found very high pre-dose and post-dose atorvastatin concentrations during treatment continuation (up to day 4), with median values of 66 (29-101) and 142 (96-237) ng/mL, respectively. Six of the nine patients explored were receiving known cytochrome P450 3A4 inhibitors (including midazolam, hydrocortisone, amiodarone, and tacrolimus; Table 6). These patients exhibited higher atorvastatin concentrations as compared with those not receiving such inhibitors: 70 (57-105) vs. 29 (27-35) ng/mL for pre-dose concentration (*P *= 0.05) and 199 (134-255) vs. 96 (80-99) ng/mL for post-dose concentration (*P *= 0.04), respectively (Figure 4).

**Table 6:** Individual characteristics of nine critically ill septic patients with atorvastatin plasma concentrations assessment during treatment continuation

Patient	Age	Diagnosis	SAPS II	ICU survivor	CA	MV	Feeding	Cytochrome P450 3A4 inhibitors
1	89	Undocumented sepsis	46	Yes	Yes	No	Oral	No
2	64	Surgical site infection	40	Yes	Yes	Yes	OGT	No
3	77	Pneumonia	44	Yes	No	No	Oral	No
4	74	Pneumonia	56	No	Yes	Yes	OGT	Yes (amiodarone, midazolam, hydrocortisone)
5	81	Cholecystitis	113	No	Yes	Yes	OGT	Yes (midazolam, hydrocortisone)
6	62	Pneumonia	54	No	Yes	Yes	OGT	Yes (hydrocortisone)
7	60	Urosepsis	35	Yes	Yes	No	Oral	Yes (tacrolimus, hydrocortisone)
8	56	Urosepsis	81	Yes	Yes	Yes	OGT	Yes (midazolam)
9	67	Liver abscess	67	Yes	Yes	Yes	OGT	Yes (amiodarone, midazolam, hydrocortisone)

CA, catecholamine therapy; MV, mechanical ventilation; OGT, oro-gastric tube; SAPS, simplified acute physiology score.

## Discussion

Our study suggests that the lesser morbidity associated with continuation of ongoing statin therapy (as compared with systematic discontinuation) in patients with severe sepsis or septic shock may be influenced by confounders. We did not find clear evidence of poor clinical tolerance of statins, but the plasma concentrations achieved during continuation of atorvastatin were particularly high.

A potential beneficial effect of statins during sepsis has been suggested by several studies reporting both a preventive effect on the risk of severe sepsis, as well as a reduction of morbidity and mortality associated with sepsis [4,17-19], but with significant heterogeneity among studies and potential publication bias [4]. The potential effect of the introduction of statins in sepsis will be resolved by currently ongoing clinical trials (NCT00528580, NCT00676897, NCT00452608, NCT00979121, NCT00450840 and NCT00357123) [20]. However, few publications have studied the effect of the continuation or discontinuation of statins during severe sepsis in patients chronically treated with statins.

In our study, patients in whom statins were continued seemed to have a better outcome compared with the discontinuation group after crude analysis. Kruger et al previously reported a particularly high mortality in bacteriemic patients in whom chronic statin therapy had been interrupted at time of the septic episode [21]. In these patients, the poorer outcomes in the discontinuation group could be due to a possible rebound effect of statins interruption on inflammatory response [22-24], but many potential sources of bias not addressed may confound the interpretation of these results [21].

In our study, there were significant imbalances between groups that may explain the differences in outcomes. In particular, patients in whom statins were discontinued had a higher prevalence of hospital-acquired infections and septic shock at ICU admission as compared with others. It is possible that more severely ill patients, and those with more complex presentation, might have been less likely to have their statins continued because their physicians were more focused on treating immediately life-threatening problems or implementing complex diagnostic procedures. After controlling for these selection bias via propensity matching and multivariable adjustment, there was no significant association between statin continuation and the main outcome (organ failure-free days). These negative findings are in line with a recent randomized trial that did not provide evidence of any beneficial role of continuing pre-existing statin therapy on sepsis progression and inflammatory parameters [25].

The absorption and metabolism of statins may vary widely in ICU patients, especially in those with sepsis, because of frequent alterations of the digestive tract function. However, only two patients in our study had treatment interrupted due to gastric intolerance. We found very high atorvastatin concentrations in patients continuing this drug, with a nearly 20-fold increase in pre-dose concentrations as compared with residual concentrations reported in healthy volunteers [26]. These results are in accordance with those of Kruger et al [26] who recently reported similarly high plasma concentrations of atorvastatin in ICU septic patients. In the later report, the peak and residual statin concentrations averaged 84 and 23 ng/mL, respectively, in septic ICU patients after a single 20 mg atorvastatin dose. Our report demonstrates even higher residual concentrations (up to 100 ng/mL) after several days of statin continuation in septic ICU patients. These high concentrations could be explained by specific pharmacokinetic and pharmacodynamic considerations in septic conditions, including increased capillary permeability, changes in plasma protein binding, and altered liver metabolism by cytochrome systems. Concomitant treatments by cytochrome P450 3A4 inhibitors may also impair atorvastatin metabolism [27,28]. Accordingly, we found significantly higher atorvastatin concentrations in patients receiving such inhibitors as compared with others.

Severe infection is a theoretical contraindication to statin administration, because these drugs might increase the risk of rhabdomyolysis and neuromyopathy [29]. We did not observe significantly more adverse events in the continuation group as compared with the discontinuation group, suggesting an acceptable tolerance of statins in the context of severe sepsis. However, aminotransferases and CPK were not systematically assessed in all patients. In addition, specific evaluation of ICU acquired myopathy was not carried out and a lack of elevated levels of circulating CPK does not rule out structural muscle injury in patients treated with statins [30]. Finally, the very high plasma atorvastatin concentrations during continuation of this drug may raise concern. Dosage regimens specifically adapted to critically ill septic patients, with particular attention to drugs susceptible to metabolic interactions, may need to be studied.

Our study has some limitations. First, the number of patients included was small, a situation that increases the chance of both type 1 and type 2 errors. Secondly, the design was retrospective, and, despite propensity matching and multivariable adjustment, the retrospective cohort design entails a number of residual biases that cannot be controlled for. Finally, although the pleiotropic effects of statins may be observed on longer term [31], our analysis was limited to the short term.

## Conclusions

In conclusion, we found that the apparent beneficial effects of continuation of chronic statin therapy in septic ICU patients were driven in part by selection bias and confounders. Although there was no clear clinical evidence of poor tolerance of statins, the very high plasma concentrations achieved during continuation of atorvastatin suggest that caution should prevail if statins are prescribed to septic patients, and their risk/benefit ratio assessed carefully.

## Key messages

• Patients in whom statin therapy had been continued in the ICU during severe sepsis or septic shock had significantly more organ failure-free days as compared with those with statin discontinuation, but this difference did not persist after propensity score matching and multivariable adjustment.

• The predose and postdose atorvastatin concentrations were particularly high in septic patients continuing the drug in the ICU. These very high concentrations advocate some caution when administering statins to septic patients in the ICU setting.

## Abbreviations

CPK: creatine phosphokinase; SAPS: simplified acute physiology score; SOFA: sequential organ failure assessment; ULN: upper limit of normal.

## Competing interests

The author(s) declare that they have no competing interests.

## Authors' contributions

AMD participated in the conception and design of the study, helped to perform the statistical analysis, and drafted the manuscript. IO participated in collection of data, helped to perform the statistical analysis, and helped to draft the manuscript. NR and BB participated in collection of data and helped to draft the manuscript. CB and AH carried out atorvastatin pharmacokinetics and helped to draft the manuscript. SK helped to perform the statistical analysis and helped to draft the manuscript. CBB participated in the conception, design and coordination of the study, and helped to draft the manuscript. All authors read and approved the final manuscript.
